# Effect of Y^3+^ Doping on Microstructure and Magnetic Transition of CuCrO_2_ Ceramics

**DOI:** 10.3390/ma18081827

**Published:** 2025-04-16

**Authors:** Haibo Lin, Shanshan Ye, Guozhu Xiong, Kailai Zhang, Yijing Su, Kan Lu, Wen Deng, Shoulei Xu, Dingkang Xiong

**Affiliations:** 1 School of Physical Science and Technology, Guangxi University, 100 East Daxue Road, Nanning 530004, China; 2 State Key Laboratory of Featured Metal Materials and Life-Cycle Safety for Composite Structures, Nanning 530004, China

**Keywords:** CuCrO_2_, Y doping, microstructure, magnetic properties

## Abstract

Ceramic samples of CuCr_1−x_Y_x_O_2_ (x = 0–0.02) were synthesized via the high temperature solid-state reaction method, and the influence of Y^3+^ doping on their microstructure and antiferromagnetic phase transitions was systematically investigated. Y^3+^ doping increased the unit cell volume from 130.928 Å^3^ for x = 0 to 131.147 Å^3^ for x = 0.0200, and the average grain size decreased from 3.38 μm for x = 0 to 4.27 μm for x = 0.0200. The Cr and Y elements maintained +3 valence, while the Cu element had +1 valence. All samples showed obvious paramagnetism when the temperature was higher than 140 K. When the temperature continued to decrease, the lattice expansion changed the bond length and bond angle of the Cr-O-Cr bond, resulting in a change in the superexchange interaction, and the magnetic susceptibility increased significantly, gradually showing antiferromagnetism. The T_N_ of the undoped sample was about 46 K, the T_N_ of the doped sample with x = 0.0175 was about 21 K, and the T_N_ of other doped samples was about 30 K. This result indicates that Y^3+^ doping enhanced the antiferromagnetism of the sample but also weakened its antiferromagnetic stability.

## 1. Introduction

Multiferroic materials, characterized by coexisting ferromagnetic, ferroelectric, or ferroelastic orders with cross-coupled interactions, have emerged as promising candidates for next-generation magnetoelectric devices and advanced information technologies due to their unique multifunctional responses [1,2,3]. Since Curie’s pioneering proposal of intrinsic magnetoelectric coupling in 1894, extensive research has focused on two principal categories: single-phase and composite multiferroics [4]. The ABO_2_-type delafossite materials belong to the Type-II single-phase multiferroic materials, such as CuCrO_2_. CuCrO_2_ emerges as a particularly promising p-type transparent conductive oxide candidate owing to its inherent hole-dominated conductivity and exceptional visible-light transmittance, positioning it as a frontrunner for thermoelectric sensors, solar energy harvesting devices, and advanced display technologies [5,6,7,8]. A comparative analysis within Type-II single-phase multiferroic materials revealed that Cr-O bonding exhibits the strongest covalent character among B-site cations, establishing chromium as the optimal B-site constituent [9,10].

The delafossite compound CuCrO_2_ crystallizes as a triangular antiferromagnetic spin-lattice structure, adopting the R-3m space group and C3v point group symmetry at ambient conditions [11]. Its layered architecture comprises alternating Cu-O-Cu dumbbell configurations along the c-axis and edge-sharing CrO_6_ octahedra, collectively forming the characteristic delafossite framework. This geometrically frustrated triangular lattice configuration inherently hosts spin frustration, driving unconventional magnetic phase transitions whose microscopic mechanisms remain subjects of active investigation. The different states, synthesis methods, and microstructures of the materials can significantly affect their physical properties [12,13,14,15,16]. Ionic substitution has emerged as an effective strategy for modulating these magnetic functionalities [17,18,19]. Elkhouni et al. demonstrated that Zn^2+^ substitution induces spin dilution through localized magnetic moment suppression, thereby constraining antiferromagnetic domains. Conversely, Ti^2+^ doping triggers a dimensional crossover from anisotropic 3D antiferromagnetic (3D-AF) magnons to 2D-AF magnetic excitations [20,21]. A study by Gao revealed that growth-oriented lattice stresses during synthesis promote preferential c-axis orientation [22]. The coexistence of Fe^3+^ and Cr^3+^ introduces hole-mediated Fe^3+^-Cr^3+^ superexchange interactions, generating ferromagnetic ordering with progressively diminished saturation magnetization as the Fe content increases. Zhang et al. also successfully synthesized Sn^4+^-substituted CuCr_1−x_Sn_x_O_2_ (x = 0–0.05) ceramics via solid-state reaction, observing enhanced unit cell volumes without altering Cu^+^/Cr^3+^ oxidation states, while significantly modifying magnetic hysteresis characteristics [23].

These studies showed that CuCrO_2_ delafossite material has rich and complex physical properties, and the study of its microstructure is helpful to understanding the magnetoelectric coupling effect with great application potential derived from its unique magnetic structure. However, the magnetic transition mechanism behind CuCrO_2_ ceramics is still unclear, and further research is needed. Rare earth ion doping is less commonly used in the existing research to study its effect on the magnetic properties of CuCrO_2_ ceramic materials. Therefore, Y^3+^ was selected as the doping element in this paper. On the one hand, Y^3+^ and Cr^3+^ have the same valence state, and Y^3+^ has no magnetism, which can avoid the too-complex influence on the magnetism of Y^3+^. On the other hand, the radius of Y^3+^ is larger than that of Cr^3+^, which allows us to study its influence on the microstructure and magnetic transformation of CuCrO_2_ ceramics while minimizing the interference of other factors and explore the possible correlation. Therefore, in this paper, a series of Y^3+^-doped ceramic samples were prepared by the high-temperature solid-state reaction method using non-magnetic rare earth ions Y^3+^ to replace part of Cr^3+^. X-ray diffraction (XRD), Raman spectroscopy (Raman), scanning electron microscopy (SEM), X-ray photoelectron spectroscopy (XPS), and the physical property measurement system (PPMS) were used to systematically study the effect of Y^3+^ doping on the lattice structure, element valence state, microstructure, and magnetic properties of CuCrO_2_ with as little magnetic influence as possible. The correlation between the microstructure and magnetic transition was obtained, which provided theoretical and experimental evidence for its magnetic transition mechanism.

## 2. Materials and Methods

A series of CuCr_1−x_Y_x_O_2_ (x = 0–0.02) ceramic samples was fabricated via the high-temperature solid-state reaction method using high-purity CuO (99.5%), Cr_2_O_3_ (99.9%), and Y_2_O_3_ (99.9%) nanopowders. The three powders were stoichiometrically weighed, and raw material powders with different doping concentrations were obtained by adjusting the ratio of Cr_2_O_3_ and Y_2_O_3_ powders. The three raw material powders were mixed and poured into an appropriate amount of alcohol so that the powder could be completely dispersed into the alcohol, then stirred with an electric stirrer for 24 h to mix the powder evenly. The mixed powder was placed in a drying oven at 85 °C for 24 h. After the alcohol was completely evaporated, the powder was ground for 30 min to make the powder fine enough. The powder was then calcined in a tube furnace in an argon atmosphere (20 mL/min) at a temperature of 900 °C for 12 h. After cooling, the powder was ground again to make the powder fine enough, and it was pressed under a pressure of 12 MPa to make a disc with a diameter of 15 mm. The final ceramic sample was obtained by calcining it in a tubular furnace in an argon atmosphere (20 mL/min) and at a temperature of 1300 °C for 12 h. The synthesized CuCr_1−x_Y_x_O_2_ (x = 0–0.02) ceramics were systematically characterized for their microstructure and magnetic properties.

The phase composition of the CuCr_1−x_Y_x_O_2_ (x = 0–0.02) ceramics was characterized using a DX-2700A X-ray diffractometer (Dandong HaoYuan Instrument, Dandong City, Liaoning Province, China in English) equipped with Cu-Kα radiation (λ = 1.5418 Å). The XRD data were fitted by GSASII software (version: 3fc6dc from 14 May 2024 14:57), and the changes in the unit cell parameters and crystal density were analyzed. The tube voltage and tube current of the diffractometer were 40 kV and 30 mA, respectively. The step angle was 0.02°, the sampling time was 0.6 s, and the test angle range was 20–80°. Vibrational characteristics were probed using an iHR550 Raman spectrometer (Horiba, South District, Kyoto, Japan) with 532 nm laser excitation, mapping phonon mode modifications across the doping concentrations. The fracture surface microstructure and grain boundary evolution were analyzed through a JSM-6510A analytical scanning electron microscope (JEOL, Toshima City, Tokyo, Japan) operated at 30 kV with the fracture surface magnified 1500 times. Surface chemical states were quantified by ESCALAB Xi+ XPS (Thermo Fisher Scientific, Waltham, Massachusetts, USA), which used a monochromatic X-ray source, with energy calibration referencing the adventitious C 1 s peak at 284.8 eV. Magnetic properties were investigated using the VSM module of a comprehensive physical properties measurement system (Quantum Design, San Diego, California, USA). The temperature dependence of the magnetic susceptibility of the samples in the temperature range of 4 K–290 K was measured under an external magnetic field of 500 Oe. The hysteresis loops at different temperatures (4 K, 26 K, 50 K, 135 K, 145 K, 290 K) were also tested.

## 3. Results and Discussion

### 3.1. XRD Spectra

**Figure 1:**
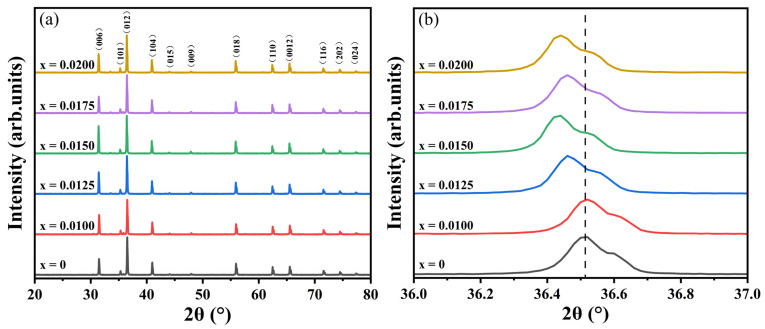
(**a**) XRD patterns of CuCr_1−x_Y_x_O_2_ (x = 0–0.02) ceramic samples and (**b**) their local amplification.

The XRD patterns of the CuCr_1−x_Y_x_O_2_ (x = 0–0.02) ceramics are presented in Figure 1a. All diffraction peaks exhibit sharp and well-defined profiles, indicative of highly crystalline sintered samples. No impurity phases associated with Y-containing compounds were detected, confirming the phase purity of the delafossite CuCrO_2_ structure (PDF # 04-010-3330), which verifies the successful incorporation of Y^3+^ into the host lattice without altering its crystallographic phase. A magnified view of the (012) diffraction peak is shown in Figure 1b. Minimal peak shifts are observed for low doping levels (x = 0.0100), suggesting negligible lattice distortion at these concentrations. However, for x > 0.0100, systematic peak shifts toward lower angles occur, signifying progressive lattice expansion with increasing Y^3+^ content. Notably, the (012) peak position shifts monotonically to lower angles at x = 0.0125 and 0.0150, consistent with the larger ionic radius of Y^3+^ compared to Cr^3+^. At higher concentrations (x = 0.0175 and 0.0200), however, a non-monotonic shift pattern emerges: initial displacement to higher angles followed by a return to lower angles. This anomaly implies an optimal doping threshold at x = 0.0150, beyond which lattice distortion becomes dominant. Such behavior likely originates from point defect formation (cation vacancies or oxygen vacancies) that disrupts long-range crystallographic ordering.

**Figure 2:**
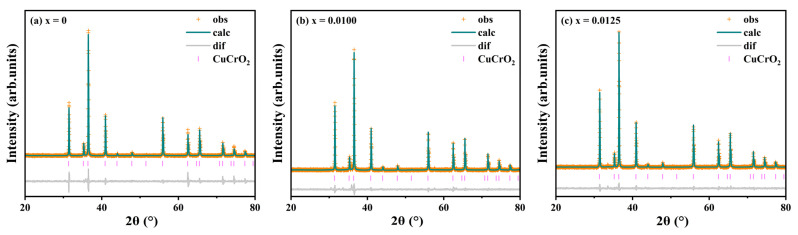

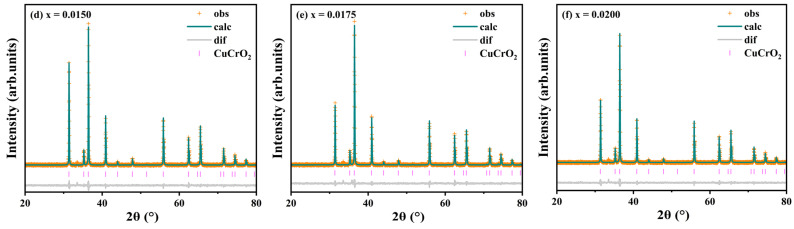
XRD refinement results for CuCr_1−x_Y_x_O_2_ (x = 0–0.02) ceramic powder samples.

Rietveld refinement of the diffraction data was performed using GSASII software. The results are shown in Figure 2 and Table 1. The R_P_ parameters of all samples are less than 10%, and the GOF parameters are less than 2, which proves that the refinement results are reliable. The refinement results shown in Table 1 show that Y^3+^ doping leads to lattice expansion (from 130.928 Å^3^ for x = 0 to 131.147 Å^3^ for x = 0.0200) and a decrease in density (from 5.6758 g·cm^−3^ for x = 0 to 5.6087 g·cm^−3^ for x = 0.0200). This is consistent with the expected lattice structure change caused by large-radius Y^3+^ doping, indicating that Y^3+^ successfully entered the lattice and replaced Cr^3+^. In addition, the c/a values of the doped samples are smaller than those of undoped samples, indicating that there is a clear preferential growth orientation in the doped samples.

The structural analysis confirms the successful substitution of Cr^3+^ by Y^3+^ in the CuCrO_2_ lattice without secondary phase formation, preserving the delafossite structure (PDF # 04-010-3330). Low Y^3+^ doping (x = 0.0100) minimally perturbs the lattice structure. However, for x > 0.0100, lattice expansion becomes evident. The optimal doping threshold is identified at x = 0.0150, beyond which lattice distortion emerges. Despite these distortions, the overall structural integrity remains preserved, as evidenced by the absence of secondary phases and maintained crystallographic stability.

### 3.2. Raman Spectroscopy

**Figure 3:**
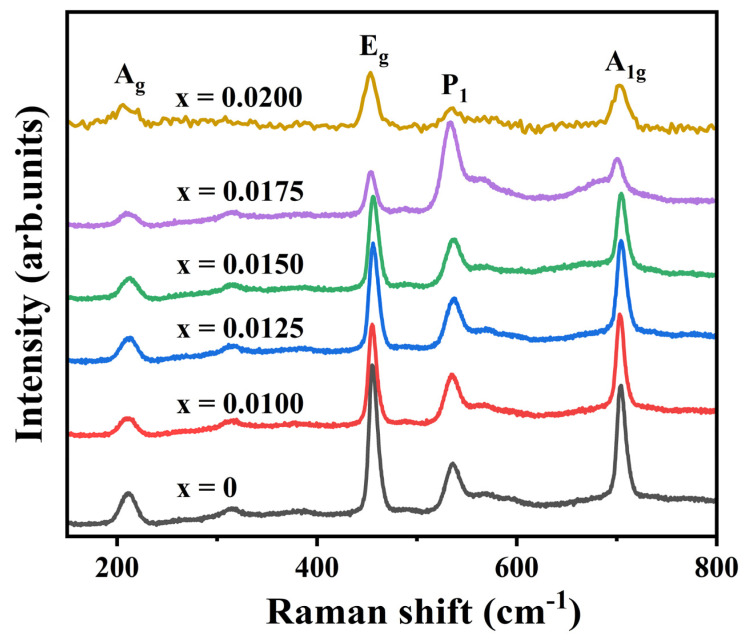
Raman patterns of CuCr_1−x_Y_x_O_2_ (x = 0–0.02) ceramic samples.

To further investigate the impact of Y^3+^ doping on the lattice structure of CuCrO_2_, Raman spectroscopy was conducted on the samples, as illustrated in Figure 3. Four distinct vibrational bands are observed across all compositions: A_g_ (208 cm^−1^), E_g_ (455 cm^−1^), P_1_ (533 cm^−1^), and A_1g_ (704 cm^−1^), consistent with prior studies [24,25]. The A_1g_ phonon mode corresponds to atomic vibrations along the Cu-O bond direction, while the E_g_ mode arises from vibrations perpendicular to the Cu-O bonds. The P_1_ mode originates from the native intrinsic acceptor point defects in CuCrO_2_ [26]. These spectral features confirm that Y^3+^ doping does not change the delafossite structure of CuCrO_2_. However, increasing the Y^3+^ concentration induces progressive attenuation of the E_g_ and A_1g_ mode intensities, accompanied by frequency downshifts. In contrast, the P_1_ mode exhibits enhanced intensity until x = 0.0200, where its relative intensity normalizes, albeit with a significantly broadened full width at half-maximum. These observations indicate that Y^3+^ doping substantially modulates phonon interactions and molecular polarization in CuCrO_2_ ceramics [27,28], weakening (Cr, Y)-O bonding. However, Y^3+^ doping does not change the delafossite structure of CuCrO_2_, which is consistent with the results of XRD.

### 3.3. Scanning Electron Microscopy

**Figure 4:**
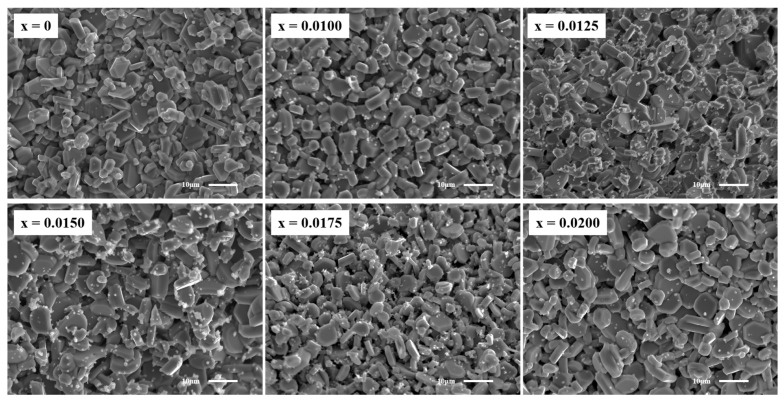
SEM images of CuCr_1−x_Y_x_O_2_ (x = 0–0.02) ceramic samples with fracture surfaces magnified 1500 times.

**Figure 5:**
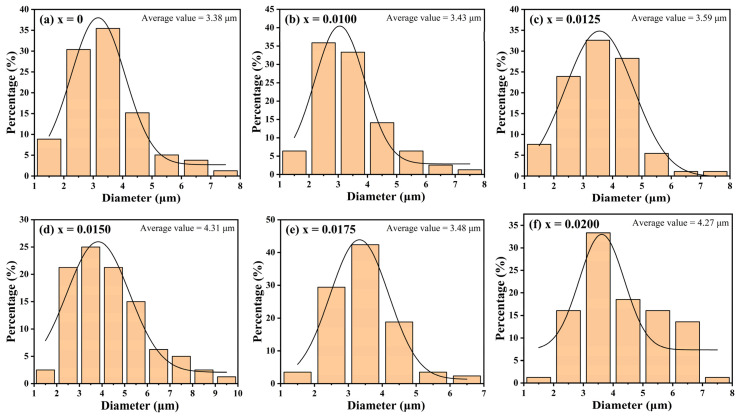
CuCr_1−x_Y_x_O_2_ (x = 0–0.02) ceramic samples’ grain size distribution bar graphs.

SEM images of the fracture surfaces of CuCr_1−x_Y_x_O_2_ (x = 0–0.02) ceramics are presented in Figure 4. The undoped sample exhibits a smooth surface with well-defined interfaces, characteristic of a layered hexagonal structure. Pores formed during synthesis due to oxygen release are observed [29,30]. In contrast, Y^3+^-doped samples display a gradual transition from hexagonal-phase to amorphous-phase growth, accompanied by blurred grain boundaries. To further analyze the effect of Y^3+^ doping on the grain size, the SEM images were analyzed by ImageJ software (version: ij154-win-java8) and the average particle size was obtained. The results are shown in Figure 5. The results show that doping significantly changes the grain size of the samples. The average grain size of all doped samples is larger than that of undoped samples, and with an increase in the doping concentration, it shows a linear growth trend (except for the sample with x = 0.0175). The results of the particle size analysis are consistent with the XRD diffraction pattern, which further confirms that Y^3+^ doping leads to lattice expansion and a density reduction, hindering the synthesis of dense CuCrO_2_ ceramics. Furthermore, Y^3+^ incorporation impedes intergranular fusion, promoting the formation of small precipitates through liquid-phase recrystallization. The content of precipitates reaches the maximum when x = 0.0150. In summary, the SEM results confirm the significant effect of Y^3+^ on the surface microstructure of CuCrO_2_ ceramics.

### 3.4. X-Ray Photoelectron Spectroscopy

**Figure 6:**
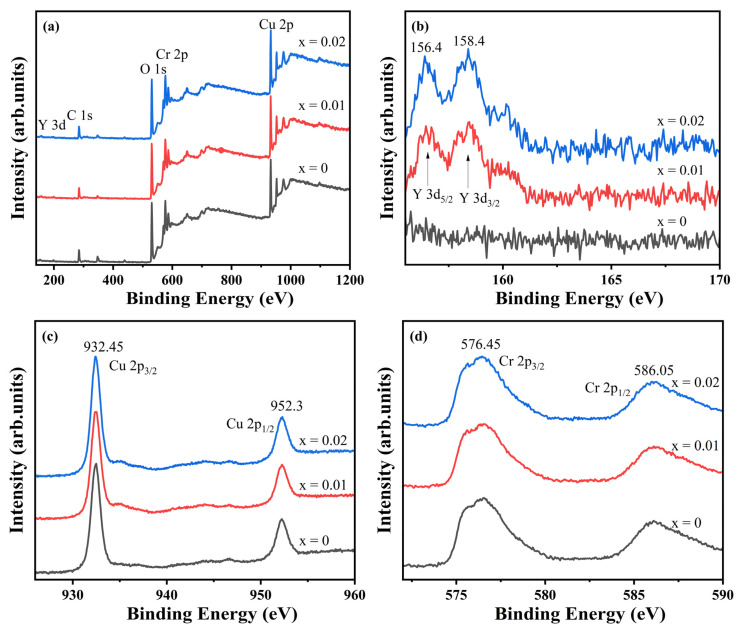
XPS spectra of CuCr_1−x_Y_x_O_2_ (x = 0, x = 0.01, and x = 0.02) ceramic samples: (**a**) survey spectra, (**b**) Y 3d, (**c**) Cu 2p, and (**d**) Cr 2p.

To investigate the influence of doping on elemental valence states, X-ray photoelectron spectroscopy (XPS) was conducted on CuCr_1−x_Y_x_O_2_ (x = 0, x = 0.01, and x = 0.02) ceramic samples. The survey spectra and high-resolution Y 3d, Cu 2p, and Cr 2p spectra are shown in Figure 6a–d. The undoped sample exhibits XPS signals exclusively from C, O, Cr, and Cu, with no detectable impurities. In contrast, Y signals emerge in the doped compositions (x = 0.01 and 0.02), confirming successful Y incorporation as designed. Carbon detection across all samples originates from ambient surface contamination. The Y 3d spectra (Figure 6b) display two sharp, symmetric peaks at 156.4 eV (Y 3d_5/2_) and 158.4 eV (Y 3d_3/2_), where Y 3d_5/2_ indicates that the Y element is +3 valence [31]. The Cu 2p spectra (Figure 6c) reveal characteristic peaks at 932.45 eV and 952.30 eV, corresponding to Cu^+^ [32,33], with no satellite features near 940 eV that would indicate Cu^2+^. Similarly, the Cr 2p spectra (Figure 6d) exhibit peaks at 576.45 eV (2p_3/2_) and 586.05 eV (2p_1/2_), confirming Cr^3+^ valence states [34,35]. These findings collectively demonstrate preserved +3 oxidation states for Y and Cr alongside +1 valence for Cu. Combined with prior structural analyses, this confirms single-phase CuCrO_2_ formation where Y^3+^ substitutes for Cr^3+^ in the lattice without inducing valence state alterations in the constituent elements, despite the observed lattice expansion effects [36].

### 3.5. Magnetic Properties

**Figure 7:**
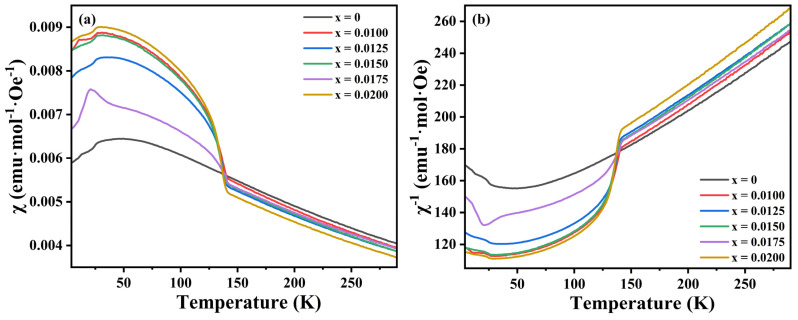
(**a**) Temperature dependence of magnetic susceptibility (χ) of CuCr_1−x_Y_x_O_2_ (x = 0–0.02) ceramic samples and (**b**) reciprocal susceptibility (χ^−1^) versus T.

Figure 7a illustrates the temperature-dependent magnetic susceptibility χ of CuCr_1−x_Y_x_O_2_ (x = 0–0.02) ceramic samples across 4–290 K. For the undoped specimen, χ demonstrates a linear enhancement with decreasing temperature above 50 K, reaching a maximum value that signifies paramagnetic behavior. Upon further cooling, a progressive reduction in χ emerges, accompanied by a transition from paramagnetic to antiferromagnetic characteristics. The Néel temperature T_N_ of the undoped sample was determined as ~46 K, which exhibits elevation compared with previous reports [17,37]. This observation suggests that the 1300 °C sintering temperature critically modifies the low-temperature magnetic properties of CuCrO_2_, effectively enhancing T_N_ and stabilizing the antiferromagnetic order. In contrast, Y^3+^-doped specimens manifest distinctly different χ evolution trends, indicating that Y^3+^ substitution substantially alters the magnetic ground state of CuCrO_2_.

A distinct linear enhancement of χ with decreasing temperature above 140 K is observed across the doped samples, indicative of a characteristic paramagnetic response. Below this critical temperature, χ increases sharply and no longer increases linearly. After reaching the peak value, the magnetic susceptibility decreases gradually again, showing obvious antiferromagnetism. The χ-T curve of the doped sample shows that near 140 K, due to the introduction of non-magnetic Y^3+^, lattice expansion or even distortion occurs, which destroys the long-range antiferromagnetic order; this results in some magnetic moments that cannot be completely offset, forming short-range order or spin tilt, so the doped sample exhibits higher magnetic susceptibility. The x = 0.0175 sample exhibits T_N_ ~21 K, while other doped counterparts demonstrate T_N_~30 K, with an additional distinctive peak emergence at 11 K for the x = 0.0100 specimen. Notably, the doped systems display marked suppression of T_N_ when compared to undoped CuCrO_2_. These findings indicate that Y^3+^ doping weakens the antiferromagnetic stability of CCO ceramics at low temperatures, which changes the original magnetic configuration and interaction of spin-triangular-lattice CuCrO_2_ ceramics, thus forming new magnetic transition points.

The Curie–Weiss temperatures (T_θ_) derived from a linear regression analysis of χ^−1^-T curves (Figure 7b) within the paramagnetic regime are summarized in Table 2. All specimens exhibit negative T_θ_ values, and Y^3+^ doping leads to a decrease in T_θ_. Except for the x = 0.0150 and x = 0.0175 compositions, the T_θ_ value gradually decreases with an increase in the doping concentration. This means that the antiferromagnetic interaction mainly exists in CuCr_1−x_Y_x_O_2_ (x = 0–0.02) ceramics, and the antiferromagnetic interaction mainly comes from the superexchange interaction of Cr-O-Cr, so an increase in the doping concentration enhances this effect.

**Figure 8:**
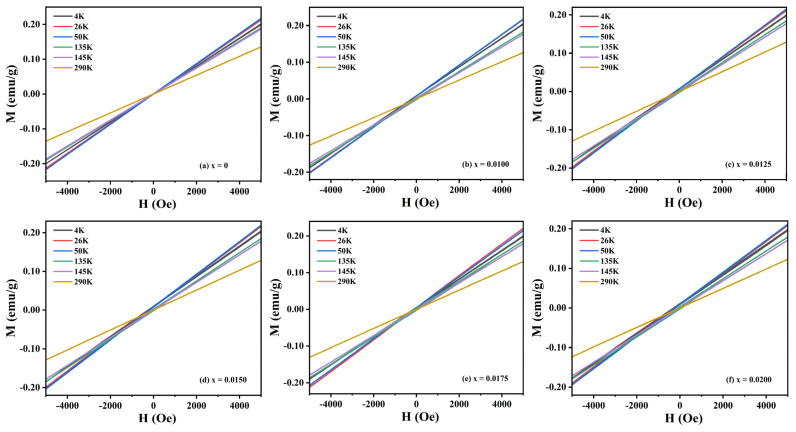
M-H curves of CuCr_1−x_Y_x_O_2_ (x = 0–0.02) samples at 4 K, 26 K, 50 K, 135 K, 145 K, and 290 K.

To elucidate the influence of Y^3+^ doping on the magnetic characteristics of CuCrO_2_ ceramics, Figure 8 presents magnetic hysteresis loops measured under a ±5000 Oe applied field at different temperatures (4 K, 26 K, 50 K, 135 K, 145 K, and 290 K). All specimens exhibit linear M-H relationships without magnetic saturation, differing solely in their slope magnitudes. Compared with that for the undoped sample, the change in the hysteresis loop slope increases obviously when the doped sample decreases from 145 K to 50 K. The curve slopes of 26 K and 50 K are the largest in all samples, corresponding to T_N_, which is consistent with the trend of the χ-T curve. It can be seen that all samples are paramagnetic at 290 K and exhibit obvious antiferromagnetism at other temperatures. As the temperature drops to 26–50 K, the magnetic susceptibility reaches its maximum, and no ferromagnetic phase appears across the entire temperature range.

The collective evidence demonstrates that Y^3+^ doping profoundly modifies the magnetic transitions in CuCr_1−x_Y_x_O_2_ (x = 0–0.02) ceramics. While the lattice expansion and structural distortion induced by dopant incorporation preserve the delafossite framework and oxidation states of Cu/Cr, they induce geometric reorganization of Cr-O-Cr bonding configurations through bond angle modification. Concurrently, the non-magnetic Y^3+^ ions spatially decouple adjacent Cr^3+^ moments, creating magnetic isolation effects. The intrinsic antiferromagnetic order of spin-frustrated CuCrO_2_ multiferroic materials comes from the superexchange interaction of Cr-O-Cr. Therefore, Y^3+^ doping leads to a change in superexchange interactions, and some magnetic moments cannot be completely offset, forming a short-range order or spin tilt; thus, the doped samples show stronger antiferromagnetism but also weakened antiferromagnetic stability at low temperature.

## 4. Conclusions

A series of CuCr_1−x_Y_x_O_2_ (x = 0–0.02) ceramic samples was synthesized via the solid-state reaction method. All specimens exhibited phase-pure delafossite structures, with no secondary phases detected. Y^3+^ doping led to lattice expansion. The cell volume increased from 130.928 Å3 for x = 0 to 131.147 Å3 for x = 0.0200, the density decreased from 5.6758 g·cm^−3^ for x = 0 to 5.6087 g·cm^−3^ for x = 0.0200, and the average grain size decreased from 3.38 μm for x = 0 to 4.27 μm for x = 0.0200. Y^3+^ doping did not change the lattice structure of CuCrO_2_, but with an increase in the doping concentration, the mutual fusion between grains was also affected. Liquid-phase recrystallization made some small precipitates appear in the sample and gradually increased with an increase in the doping concentration. The content of precipitates reached the maximum in the sample with x = 0.0150. An XPS analysis confirmed monovalent Cu⁺ and trivalent Y^3+^/Cr^3+^ states, excluding divalent copper species. All samples showed obvious paramagnetism when the temperature was higher than 140 K. However, when the temperature was lower than 140 K, the lattice expansion and lattice distortion caused by Y^3+^ doping changed the bond angle of the Cr-O-Cr bond and enhanced the superexchange interaction of Cr-O-Cr, resulting in some magnetic moments not being completely offset. As a result, a short-range order or spin tilt formed, enhancing the antiferromagnetism of the sample but also weakening its antiferromagnetic stability at low temperature. The T_N_ of the undoped sample was about 46 K. The T_N_ of the sample with x = 0.0175 was about 21 K, while the T_N_ of the other doped samples was about 30 K. The sample with x = 0.0100 also had an obvious peak at 11 K. In addition, it was found that sintering at 1300 °C significantly enhanced the antiferromagnetic stability of CuCrO_2_ and increased the Néel temperature T_N_. This study confirmed that low-concentration Y^3+^ doping can fine-tune the lattice structure of CuCrO_2_ ceramics without destroying the main phase and regulate its magnetic transition, which provides a new idea for regulating and improving the magnetic properties of CuCrO_2_ by rare earth element doping.

## Figures and Tables

**Table 1:** Lattice parameters of CuCr_1−x_Y_x_O_2_ (x = 0–0.02) ceramic samples.

Sample	x = 0	x = 0.0100	x = 0.0125	x = 0.0150	x = 0.0175	x = 0.0200
a = b (Å)	2.9734	2.9761	2.9756	2.9751	2.9759	2.9755
c (Å)	17.1000	17.1086	17.1063	17.1043	17.1060	17.1045
c/a	5.7510	5.7487	5.7489	5.7492	5.7482	5.7484
Cell volume (Å^3^)	130.928	131.236	131.168	131.114	131.198	131.147
Density (g·cm^−3^)	5.6758	5.6253	5.6257	5.6124	5.6063	5.6087
R_P_ (%)	9.47	7.88	6.67	6.65	7.47	7.08
GOF	1.62	1.33	1.16	1.16	1.28	1.22

**Table 2:** Curie–Weiss temperatures for CuCr_1−x_Y_x_O_2_ (x = 0–0.02) samples.

Samples	x = 0	x = 0.0100	x = 0.0125	x = 0.0150	x = 0.0175	x = 0.0200
T_θ_ (K)	−210.04	−224.82	−252.73	−251.43	−218.09	−259.49

## Data Availability

The original contributions presented in this study are included in the article. Further inquiries can be directed to the corresponding author.
